# Impairment of Endogenous H_2_S Pathway due to Aging and Endothelium Denudation in Mouse Isolated Thoracic Aorta

**DOI:** 10.33549/physiolres.935419

**Published:** 2025-02-01

**Authors:** Fatma AYDİNOGLU, Ebru Nisa ERDEM, Tugba TOYRAN, Nuran OGULENER

**Affiliations:** 1 Department of Pharmacology, Pharmacy Faculty, Cukurova University, Adana, Republic of Türkiye; 2 Department of Pathology, Medical Faculty, Cukurova University, Adana, Republic of Türkiye; 3 Department of Pharmacology, Medical Faculty, Cukurova University, Adana, Republic of Türkiye

**Keywords:** Aging, Hydrogen sulfide, L-cysteine, Endothelium, Thoracic aorta

## Abstract

Hydrogen sulfide (H_2_S) is a gas neurotransmitter that is synthesized in various mammalian tissues including vascular tissues and regulates vascular tone. The aim of this study is to investigate whether the endogenous L-cysteine/H_2_S pathway is impaired due to aging and endothelial denudation in mouse isolated thoracic aorta. For this purpose, young (3–4 months) and old (23–25 months) mice were used in the experiments. The effects of aging and endothelium on endogenous and exogenous H_2_S-induced vasorelaxation were investigated by cumulative L-cysteine-(1 μM-10 mM) and NaHS-(1 μM-3 mM) induced vasorelaxations, respectively. The L-cysteine-induced relaxations were reduced in old mice aorta compared to the young mice. Also, vasorelaxant responses to L-cysteine (1 μM-10 mM) were reduced on aorta rings with denuded-endothelium of young and old mice. However, the relaxation responses to NaHS were not altered by age or endothelium denudation. The loss of staining of CSE in the endothelial layer was observed in old thoracic aorta. Ach-induced (1–30 μM) relaxation almost abolished in endothelium-denuded rings from both mice group. Also, relaxation Ach reduced in intact endothelium tissue of old mice aorta. In conclusion, the vasorelaxant responses to L-cysteine but not NaHS decreased and the protein expression of CSE reduced in old thoracic aorta rings consistent with a decrease in H_2_S concentration with aging and endothelium damage, suggesting that aging may be lead to decrease in enzyme expression and H_2_S signaling system due to endothelium damage in mouse thoracic aorta.

## Introduction

Hydrogen sulfide (H_2_S) is identified as an endogenous gasotransmitter such as carbon monoxide (CO) and nitric oxide (NO) [1,2]. H_2_S is synthesized by cystathionine-gama-lyase (CSE), cystathionine-beta-synthetase (CBS) and 3-mercaptopyruvate sulfur-transferase (3-MST) enzymes from L-cysteine or L-homocysteine, and it contributes to various physiological effects in many organs and tissues [3,4]. Vascular actions of H_2_S are extremely complex. Also, H_2_S induced a dual vascular effect, as vasoconstriction and vasodilation [5]. Preliminary studies reported that H_2_S cause vasorelaxation through activating ATP-sensitive potassium channels in vascular smooth muscle and it is generated only in smooth muscle cells of this tissue [6]. However, it is recently indicated that H_2_S is generated and released from endothelial cells, which induces the relaxation of vascular smooth muscle cells [4]. Endothelium is a layer of cells, which cover the internal surface of blood vessels. Endothelial dysfunction occurs in many cardiovascular diseases, which involves different mechanisms, including impaired endothelium-derived vasodilators and enhanced endothelium-derived vasoconstrictors [7]. Experimental studies have demonstrated that endothelium-dependent acetylcholine relaxation decreased in endothelium-denuded vascular smooth muscle cell [8–10].

H_2_S seems to have an essential and complex contribution to endothelium-dependent vasorelaxation. Recently, it has been reported that there is a decrease in H_2_S synthesis due to deterioration of endothelium function due to aging in human and animal models [11]. The reduced generation of endogenous H_2_S is demonstrated in hypertension, as well as in the aging process [12]. Also, H_2_S-induced vasorelaxation attenuated by removal of the endothelium in rat aortic tissues [13]. On the other hand, Predmore *et al*. reported that the augmentation of H_2_S relaxation observed in rat aorta rings with age. [14]. These contradictory findings regarding the interaction between aging and H_2_S need to be clarified.

The aim of this study is to investigate whether the endogenous L-cysteine/H_2_S pathway is impaired due to aging and endothelial denudation in mouse isolated thoracic aorta. In the present study, *in vitro* L-cysteine/NaHS-induced relaxation, immunohistochemically CSE, CBS and 3-MST protein expression and endogenous H_2_S production level were evaluated to identify the effect of aging and endothelial damage on H_2_S function in thoracic aorta of young and old mice.

## Materials and Methods

### Animals

In the present study, 3–4 months young and 23–24 months old male Swiss albino mice were obtained from Cukurova University Health Sciences Application and Research Center (SABIDAM). The experimental protocol was approved by the Local Ethics Committee of Cukurova University. Mice were kept under 12 h light/darkness cycles environmental conditions and allowed free access to food and water. Protocols were conducted in accordance with national and international guidelines for the care and use of laboratory animals and approved by the Institutional Animal Care and Use Committee of Cukurova University and given the approval number: 4/3/08.07.2021.

### Experimental protocol

Mice were killed by cervical dislocation. Descending thoracic aortas from young and old mice were carefully removed and immediately were placed in Krebs solution (mM: NaCl: 118, KCl: 5.4, CaCl_2_: 2.5, MgSO_4_: 1.2, NaHCO_3_: 25, NaHPO_4_: 25, glucose: 11.1). The aorta was divided into 3–4 mm-long segments (typically 2 segments per mouse) that were each mounted under 1 gram tension on parallel wires in a tissue bath system maintained at 37 °C and gassed with a mixture of 95 % O_2_ and 5 % CO_2_ at pH 7.4. Aortic segments were allowed to equilibrate for 60 min, during which the medium was changed every 15 min. Changes in muscle length were recorded isometrically *via* an isometric transducer (MP35).

The relaxant responses of aorta rings with endothelium (intact) and without endothelium (denuded) from young and old groups were evaluated. The aorta rings were stripped off the endothelium by gently rubbing the inner surface. The presence of endothelium was confirmed by adding acetylcholine (10 μM) to induce at least 50 % relaxation of phenylephrine (5 μM) pre-contracted rings, whereas a relaxation of 10 % indicated the aorta segments were successfully denuded.

In the first set of experiments, to evaluate the relaxant responses to endogenous H_2_S, the intact and denuded aorta rings from young and old mouse were contracted with phenylephrine (5 μM). After the contraction response reached a plateau, relaxation responses were obtained by applying cumulatively L-cysteine (1 μM-10 mM) to the tissues. After relaxation responses were obtained, tissues were incubated 30 min with Krebs solutions and second series of relaxations were record in the same manner. Also, we evaluated the relaxant responses to exogenous H_2_S on isolated intact and denuded-aorta rings from young and old mice. NaHS was used as a H_2_S donor and was freshly prepared on the day of every experiment. Following to 60 min incubation period the rings were pre-contracted by phenylephrine (5 μM). After a steady state contraction obtained, cumulative NaHS (1 μM-3 mM) was applied. After first series relaxant responses were obtained, tissues were incubated 30 min and second series of relaxations were record in the same manner. Furthermore, in young and old group, the isolated intact and denuded-aorta rings were exposed to cumulative doses of acetylcholine (1–30 μM) on phenylephrine-pre-contracted rings.

### Measurement of endogenous H_2_S release in mouse aorta rings

H_2_S production in aorta tissues was determined with a commercially available H_2_S Colorimetric Assay kit (Elabscience Biotechnology Co., Ltd, Wuhan, China) through the reaction between H_2_S and zinc acetate, N, N-Dimethyl-p-phenylenediamine, and ammonium ferric sulfate. Protein concentration was determined by using a bicinchoninic acid assay kit (Sigma Chemical Co, St. Luis, MO). Aorta tissues were homogenized in extraction solution and centrifuged for 10 min at 4 °C at 10000× g, and the supernatant was collected. The supernatant solution was mixed with an equal volume of Reagent 1 and 2. After centrifugation, the sediment was dissolved in Reagents 1, 3 and 4. The supernatant obtained after centrifugation was mixed Reagent 5. The absorbance of solutions was measured after 20 min at a wavelength of 665 nm H_2_S concentrations in aorta tissues, expressed as nmol/mg protein.

### Morphologic and immunohistochemically studies

Aorta tissues were identified in formaldehyde solution and preserved, then embedded in paraffin blocks. Sections of 5 microns thickness were obtained and stained with hematoxylin and eosin (H&E). Additionally, for immunohistochemically examination, sections of 5 microns thickness were obtained from paraffin blocks. After waiting for one hour at 60 °C, the sections were de-paraffinized with xylene for 15 min. They were then hydrated through decreasing concentrations of alcohol and washed with distilled water. The prepared sections were subjected to immunostaining with CSE (dilution 1:500, Abcam), CBS (dilution 1:50, Abcam), and 3-MST (dilution 1:1000, Sigma) antibodies. Also, liver for CSE, brain for CBS and, kidney for 3-MST were used as positive controls. The staining was performed using an automated immunohistochemistry device, the BenchMark XT, with the Ultraview DAB kit. The stained preparations in the automated staining device were covered with a liquid-based cover medium. To evaluate the staining intensity of antibodies, the expression was graded as follows: grade 0 in case of absence of staining or staining in less than 10 % of cells; grade 1 for staining in 10 % to 30 % of cells; grade 2 for staining in 31–60 % of cells and grade 3 for staining in more than 60 % of cells.

### Drugs

The following drugs were used; acetylcholine chloride, phenylephrine hydrochloride, L-cysteine, sodium hydrosulphide hydrate (Sigma Chemical Co., St. Louis, MO, U.S.A.). All drugs were dissolved in distilled water. NaHS was prepared fresh before each experiment and kept on ice.

### Statistical analysis

Cumulative L-cysteine, NaHS and Ach-induced relaxations were evaluated as a percentage of phenylephrine contractions. Emax and pEC_50_ (−log M) were expressed as the maximum relaxation achieved by L-cysteine, NaHS and Ach. All data are presented as mean ± S.E.M. Differences in results between tissues were tested by two-way analysis of variance (ANOVA). *P* values less than 0.05 were considered to be significant.

## Results

### Age-dependent relaxation of mouse thoracic aorta by L-cysteine and NaHS

**Fig. 1:**
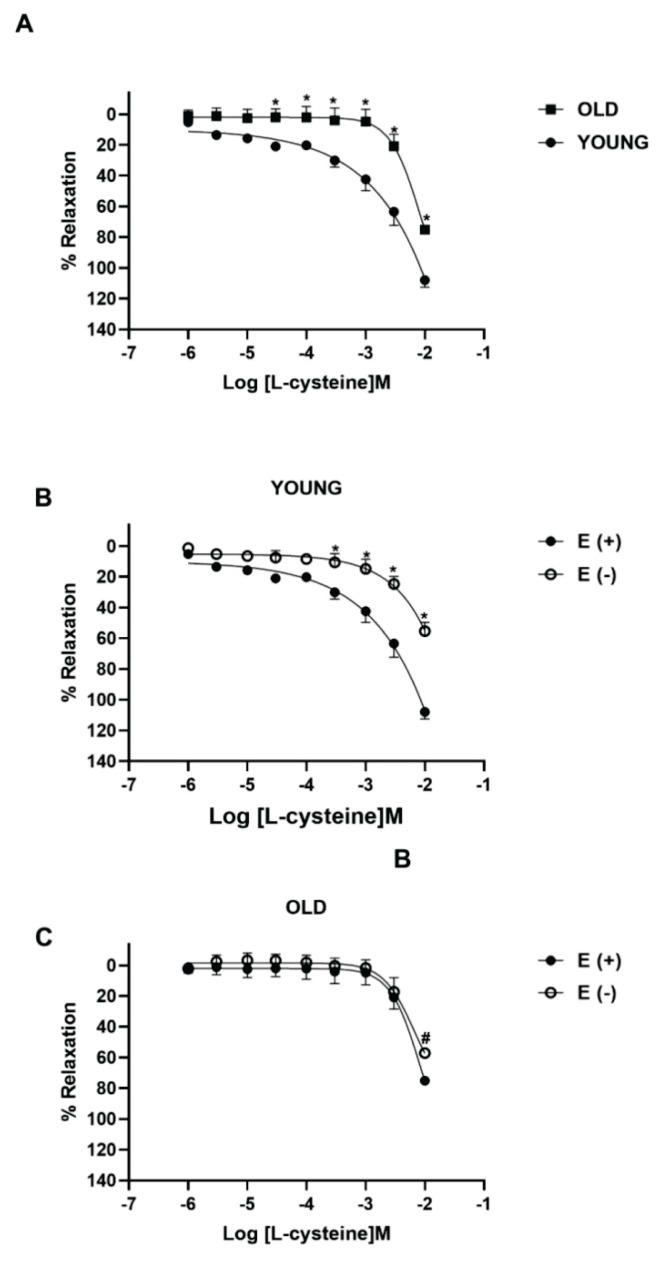
The relaxant effect of L-cysteine on isolated thoracic aorta from young and old mice. Concentration-response curves for L-cysteine (1 μM-10 mM) on thoracic aorta isolated from young and old mice (**A**), endothelium intact and denuded thoracic aorta isolated from young mice (**B**) and old mice (**C**). For each experiment, relaxant responses are expressed as percentage of the contractile responses induced by phenylephrine (5 μM). All values are mean ± S.E.M. (n=5); * *P*<0.05 significantly different from young; ^+^ *P*<0.05 significantly different from endothelium-intact young aorta rings; ^#^ *P*<0.05 significantly different from endothelium-intact old aorta rings; two-way analysis of variance (ANOVA) corrected from multiple comparisons (Bonferroni corrections).

**Fig 2:**
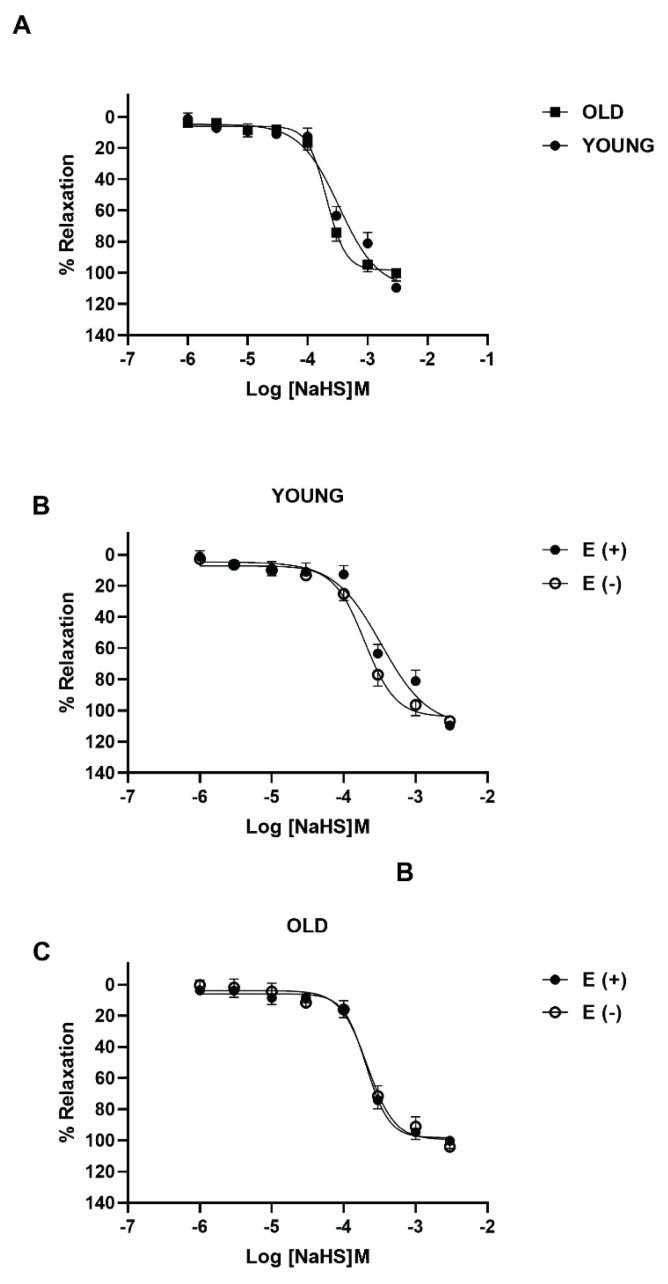
The relaxant effect of NaHS on isolated thoracic aorta from young and old mice Concentration-response curves for NaHS (1 μM-3 mM) on thoracic aorta isolated from young and old mice (**A**), endothelium intact and denuded thoracic aorta isolated from young mice (**B**) and old mice (**C**). For each experiment, relaxant responses are expressed as percentage of the contractile responses induced by phenylephrine (5 μM). All values are mean ± S.E.M. (n=5).

Isolated thoracic aorta rings from young and old mice were pre-contracted by phenylephrine (5 μM) and then allowed to relax upon cumulative addition of L-cysteine (1 μM-10 mM) and NaHS (1 μM-3 mM) as endogenous and exogenous H_2_S donor, respectively. Phenylephrine-induced contractions did not change in young and old mice aorta tissues (data not shown). L-cysteine and NaHS induced a concentration dependent vasorelaxation of phenylephrine pre-contracted mouse aorta tissues from both age groups. The maximum relaxant response (Emax) of L-cysteine were significantly decreased in old mice aorta compared to the young mice (*P*<0.05; n=5; Fig. 1A). In aorta rings from old mice, both Emax and pEC_50_ values of L-cysteine significantly decreased compared to aorta rings from young mice by the aging process (Table 1). On the other hand, there were no age-dependent differences in NaHS-induced vasorelaxant effects between young and old mice aorta segments (*P*>0.05; n=5, Fig. 2A).

### Endothelium-dependent relaxation by L-cysteine and NaHS in young and old mouse thoracic aorta

To investigate the effect of aging on endothelium-dependent H_2_S-induced vasorelaxation, we studied the relaxant responses to L-cysteine (1 μM-10 mM) on aorta rings with intact and denuded-endothelium from young and old mice. In young mice, the maximum of the relaxation to L-cysteine significantly decreased in denuded endothelium aorta rings compared to tissues with an intact endothelium (*P*<0.05; n=5; Fig. 1B). Emax to L-cysteine and pEC_50_ values were significantly decreased in denuded endothelium rings of the aorta from young mice compared to intact endothelium (*P*<0.05; Table 1). In old mice, only the Emax of L-cysteine were reduced by just removing endothelium from the aorta tissues (*P*<0.05; n=5 Fig. 1C). On the other hand, the relaxant responses to NaHS in denuded-endothelium were similar to tissues with intact endothelium from young and old mice (*P*>0.05; n=5; Fig. 2B, C). Emax) and pEC_50_ values to NaHS were not altered (Table 1). Phenylephrine-induced contractions did not change in endothelium-denuded aorta rings from both mice group (data not shown).

### Age and endothelium-dependent relaxation by Ach in young and old mouse thoracic aorta

**Fig. 3:**
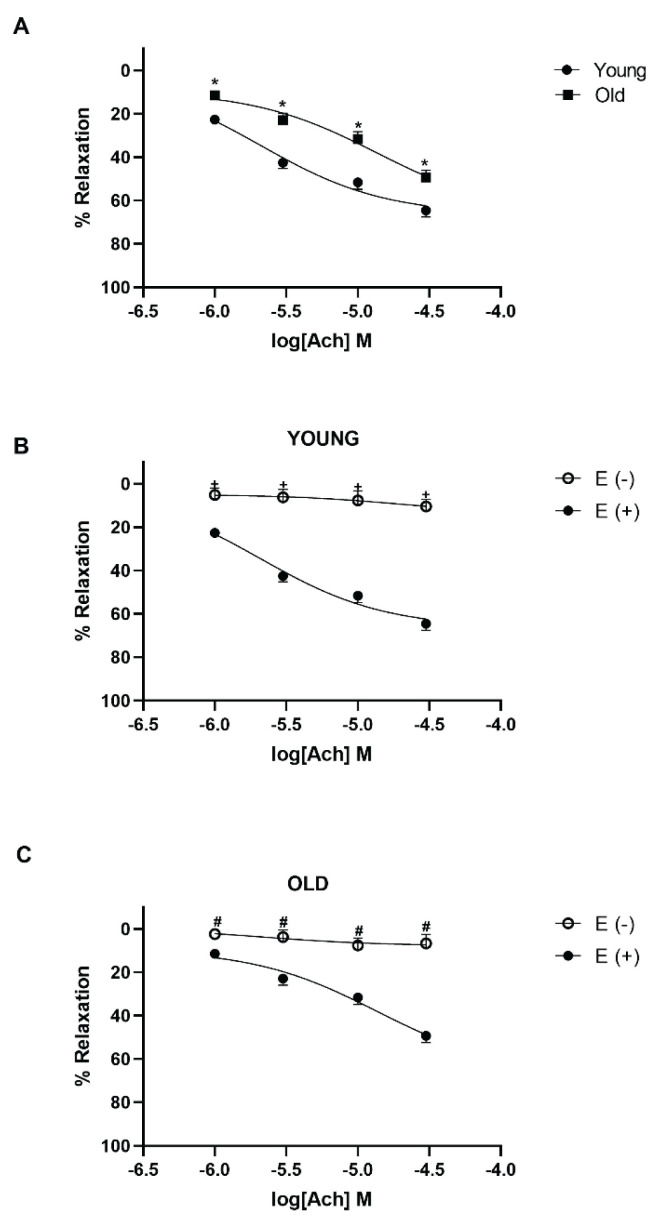
The relaxant effect of acetylcholine on isolated thoracic aorta from young and old mice. Concentration-response curves for Acetylcholine (ACh: 1–30 μM) on thoracic aorta isolated from young and old mice (**A**), endothelium intact and denuded thoracic aorta isolated from young mice (**B**) and old mice (**C**). For each experiment, relaxant responses are expressed as percentage of the contractile responses induced by phenylephrine (5 μM). All values are mean ± S.E.M. (n=5); * *P*<0.05 significantly different from young; ^+^ *P*<0.05 significantly different from endothelium-intact young aorta rings; ^#^ *P*<0.05 significantly different from endothelium-intact old aorta rings; two-way analysis of variance (ANOVA) corrected from multiple comparisons (Bonferroni corrections).

To confirm the role of aging on the endothelium-dependent relaxation, we studied the relaxant responses to Ach (1–30 μM) on aorta rings with intact and denuded-endothelium from young and old mice. In intact-endothelium aorta rings from old mice, Ach-induced maximum relaxant responses significantly diminished compared to the young mice (*P*<0.05; n=5; Fig. 3A). In aorta rings from old mice, the Emax and pEC_50_ values of Ach reduced by the aging process (Table 1). On the other hand, Ach-induced relaxation almost abolished in endothelium-denuded rings from both mice group.

### Effect of aging on the morphological structure and immunohistochemically expression of CBS, CSE and 3-MST in thoracic aorta

**Fig. 4:**
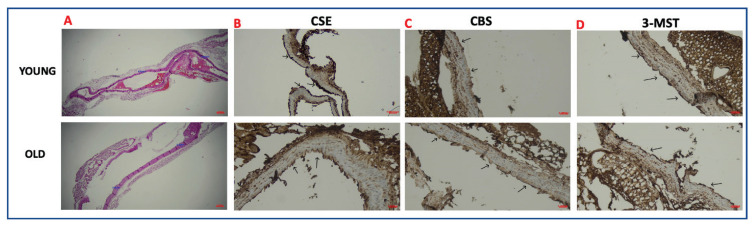
The histopathological and immunohistochemically evaluation of thoracic aorta isolated from young- and old mice. The representative images (×200) showing to hematoxylin and eosin staining of thoracic aorta from young and old mice; the thickness of vessels walls mentioned for young and old tissues (**A**). Representative images showing to the immunohistochemistry data of CSE (**B**), CBS (**C**) and 3-MST (**D**) in thoracic aorta isolated from young- and old mice. Endothelial layer (black arrow), CSE, cystathionine-gamma-lyase; CBS, cystathionine-beta-synthase; 3-MST, 3-mercaptopurivate sulphurtranspherase.

The structures of vascular tissues from young and old mice were evaluated by hematoxylin and eosin staining. Young vessel walls were thinner than old vesselwalls. However, no histopathological findings were observed in the vessels from old and young mice (Fig. 4A). For CSE antibody, it was detected that the endothelial layer was stained with diffuse cytoplasmic positive staining (Grade 3) in young vascular tissue while loss of staining (Grade 1) in the endothelial layer was observed in old thoracic aorta (Fig. 4B). For 3-MST antibody, cytoplasmic positive staining was observed in the endothelial layer of both young and old thoracic aorta, but the staining in the endothelial layer in old vascular tissue appears pale (Grade 2) (Fig. 4C). CBS antibody stained (Grade 1) in both young and elderly vascular smooth muscle cells (Fig. 4D).

In immunohistochemically studies, liver tissue for the CSE antibody, exhibiting cytoplasmic staining in hepatocytes, brain tissue for the CBS antibody, exhibiting cytoplasmic and nuclear staining and kidney tissue for the MST antibody, exhibiting cytoplasmic staining was used as a positive control.

### Effects of aging and endothelium on H_2_S generation in thoracic aorta

**Fig. 5:**
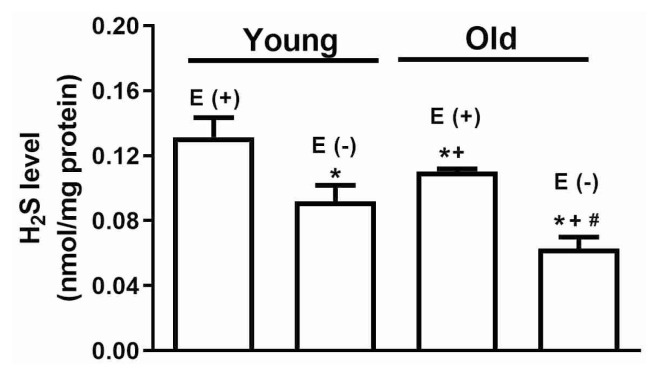
The role of aging and endothelium on basal H_2_S formation in mouse aorta tissues. The bar graph shows to the basal H_2_S levels in aorta tissues isolated from young- and old-mice groups. The all values are Mean ± S.E.M. * *P*<0.05 significantly different from endothelium-intact young-mice [E (+)]; ^+^ *P*<0.05 significantly different from endothelium-denuded young-mice [E (−)]; ^#^ *P*<0.05 significantly different from E (+) old-mice; two-way analysis of variance (ANOVA) followed by Bonferroni’s comparison test.

To evaluate that the effect of aging on basal H_2_S production was measured in thoracic aorta isolated from young and old mice. The mouse aorta generated detectable amounts of H_2_S in young and old aorta tissues. The basal H_2_S level was determined 1.19-fold less in strips of the endothelium-intact old-mice compared to endothelium-intact young group. Also, the contribution of endothelium to H_2_S production was examined in aorta tissues from young and old mice. The H_2_S was decreased by approximately 1.43-fold in the endothelium-denuded group compared to the endothelium intact young group. Also, the basal level of H_2_S was reduced 1.76-fold in the endothelium denuded old group compared to endothelium intact old group (Fig. 5).

## Discussion

In the present study, we observed that aging and endothelial dysfunction cause decreased production of endogenous H_2_S and vasorelaxation to L-cysteine due to deficiency of CSE expression, without affecting the vasorelaxant response to exogenous H_2_S in mouse thoracic aorta. These results suggest that aging may be lead to decrease in enzyme expression and H_2_S signaling system due to endothelium damage in mouse thoracic aorta.

H_2_S, a gaseous transmitter, contributes significantly to regulating cardiovascular system function and aging processes [15–17]. The maintaining of vascular tone is an important physiological function of H_2_S in the vascular system [18]. In the vasculature, endogenous H_2_S is synthesized enzymatically by CSE and 3-MPST [4,19,20]. CSE predominantly exists in the cardiovascular system while [21] CBS is present in the liver, gut, pancreas, kidney, and especially central nervous system (CNS) [1,22,23]. It has been shown that the physiological level of endogenous H_2_S has an essential role in maintaining the structural and functional integrity of vascular tissues. [24]. Also, it has been shown that H_2_S at low concentrations contracts rat mesenteric and gastric artery, while some other groups have reported that H_2_S induces relaxation responses in isolated rat vascular tissues including the aorta and the mesenteric artery *in vitro* [25,26,27,28].

Also, aging dramatically affects the function of the vascular tissue and aged-related vascular diseases are induced by vascular dysfunction [29]. H_2_S levels are reported to decrease upon aging and in age-related diseases [30]. In the present study, L-cysteine caused vasorelaxation on phenylephrine-contracted aorta rings of mice, as consistent with reports of Smimmo *et al*. and Yetik-Anacak *et al*. [31,32]. Our functional studies show that L-cysteine-induced vasorelaxation significantly reduced in thoracic aorta rings from old mice compared to young mice. In addition, the decrease of L-cysteine-induced vasorelaxant responses was observed in endothelium-denuded thoracic tissues from young mice whereas the responses did not change in endothelium intact tissue of young, suggesting that the decrease in L-cysteine vasorelaxant responses may be due to the deterioration of endothelium function due to aging. In consistent with our findings, H_2_S-induced vasorelaxation was attenuated by removal of the endothelium in rat aorta tissues [13]. Our findings indicate that, the decrease in L-cysteine-induced vasorelaxant responses may result from endothelium dysfunction that develops due to aging. In contrast, Predmore *et al*. reported that the augmentation of H_2_S relaxation observed in rat aorta rings with age [14]. The conflicting findings may be due to differences in species, age ranges, experimental protocols and gender. Also, in our study, we observed that denuded-endothelium and aging did not change phenylephrine-induced contractions in thoracic aorta rings, consistent with Fukuda *et al*. [33]. On the other hand, there are also studies that reduce or increase phenylephrine-induced contractions due to endothelium or age in thoracic aorta [34,35].

It has been shown that H_2_S is physiologically generated by CSE, and mice genetically deficient in this enzyme display marked hypertension and reduced endothelium-dependent vasorelaxation. [19–21]. Besides, the expression of CAT and 3-MST is demonstrated in rat aortic endothelial cells [4]. In the present study, the loss of CSE staining was observed in the endothelium of the old tissue compared to young mice. Also, 3-MST staining, was observed in the endothelial layer of both young and old thoracic aorta. These findings suggest that CSE may be main enzyme responsible for endogenous H_2_S release in mouse thoracic aorta endothelium and 3-MST also may play a role, and H_2_S activity decreases as a result of the decrease in the expression of these enzymes in the endothelium due to aging. In consistent with our findings, it is reported that the expression of mRNA and protein were reduced in mice by an age-dependent, and the lack of CSE caused severe aortic elastolysis and medial degeneration in aged male mice [36]. Also, CSE protein expression was downregulated in renal artery tissue in the elderly group and endothelial dysfunction associated with aging is closely related to reduced endogenous H_2_S levels and ferroptosis in vascular endothelial cells [11]. In contrast to our findings, mRNA of the CSE was detected in the smooth muscle of rat aorta, but not endothelial layer [6], and CSE protein expression increased with age in aorta of rat [14]. As a further confirmation of the aging affects endogenous H_2_S synthesis *via* enzymes, we observed that vasorelaxant responses to NaHS did not change in both old mice, and disruption of the endothelium did not affect vasorelaxation in both young and old mice, supporting that the decrease in H_2_S function due to aging occurs through enzymes. In consistent with our finding, the relaxant effect of NaHS did not alter in the endothelium-denuded preparations in mouse aorta [37]. Indeed, in the present study, H_2_S levels decreased significantly in old mice compared to young mice, and damage of the endothelium reduces H_2_S levels in both young and old mice, suggesting that H_2_S release is impaired due to aging and damage of endothelium. Also, it has been reported that age affect the hydrogen sulfide production in vessels, heart and kidneys [12].

It is well known that acetylcholine causes endothelium-dependent relaxation [38–40]. The other finding that supports our hypothesis is the inhibition of acetylcholine relaxation in intact endothelium tissue of old mice aorta rings. Ach did not produced relaxation in endothelium-denuded rings from both mice group. In consistent with our findings, Koga *et al*., reported that endothelium-dependent relaxations mediated by the muscarinic receptors are reduced with aging [41]. Also, impairment of relaxation to methacholine is demonstrated in CSE-/- mice [19]. In addition, acetylcholine-induced endothelium-dependent vasorelaxation was insensitive to NOS and COX inhibition, and this EDHF-mediated component was substantially inhibited in CSE knockout mice in mice mesenteric artery rings [42]. Further investigations are required to determine the role of H_2_S pathway and aging in acetylcholine-induced endothelium-dependent vasorelaxation.

Furthermore, it has been reported that serine derived from L-cysteine induced vasorelaxation in aorta tissue of mouse [43]. So that, the vasorelaxant effect of serine is not excluded, and further studies are needed to clarify the role of serine on the alterations of L-cysteine-induced relaxations by aging.

In conclusion, the vasorelaxant responses to L-cysteine but not NaHS decreased and the protein expression of CSE reduced in old thoracic aorta rings consistent with a decrease in H_2_S concentration with aging and endothelium damage, suggesting that aging may be lead to decrease in enzyme expression and H_2_S signaling system due to endothelium damage in mouse thoracic aorta.

## Figures and Tables

**Table 1:** Mean pEC_50_ and Emax values for the relaxant effect of L-cysteine, exogenous H_2_S (NaHS) and acetylcholine on isolated mouse endothelium intact [E (+)] and denuded [E (−)] thoracic aorta from young and old mice.

	YOUNG		OLD	

*L-cysteine*	E (+)	E (−)	E (+)	E (−)
**Emax (%)**	107.90±4.75	55.27±5.49*,+	75.00±2.69*	57.03±2.41*,+
**pEC_50_**	2.57±0.14	1.81±0.12*	1.94±0.12*	1.75±0.12*

***NaHS***				

**Emax (%)**	109.50±3.08	106.70±2.96	104.00±4.40	100.20±5.12
**pEC_50_**	3.43±0.09	3.64±0.10	3.53±0.09	3.55±0.10

***Acetylcholine***				

***Emax (%)***	64.50±3.11	10.40±3.22*,+	49.26±3.20*	6.63±4.03*,+
**pEC_50_**	5.65±0.08	3.52±0.25*,+	4.96±0.08	3.01±0.72*,+,#

Data represent mean ± S.E.M.

* *P*<0.05 significantly different from young E (+);

+ significantly different from E (+) old-mice;

# *P*<0.05 significantly different from E (−) young-mice by analysis of two-way corrected for multiple comparisons (Bonferroni corrections).
